# CMRA with 100% navigator efficiency with 3D self navigation and interleaved scanning

**DOI:** 10.1186/1532-429X-16-S1-O8

**Published:** 2014-01-16

**Authors:** Jonathan Powell, Claudia Prieto, Markus Henningsson, Peter Koken, Rene Botnar

**Affiliations:** 1 Division of Imaging Sciences and Biomedical Engineering, King's College London, London, UK; 2 Wellcome Trust and EPSRC Medical Engineering Centre, King's College London, London, UK; 3 BHF Centre of Research Excellence, King's College London, London, UK; 4 Escuela de Ingeniería, Pontificia Universidad Catolica de Chile, Santiago, Chile; 5 Philips Research, Hamburg, Germany

## Background

In recent years many novel self navigation techniques have been proposed to solve the problems associated with cardiac motion in coronary MR angiography (CMRA). A new method of interleaved scanning (iScan) allows more flexibility in designing and testing novel imaging sequences as independent scans, with different imaging parameters including k-space trajectories, can be interleaved. We compared the performance of a 3D self navigator (3DSN) with 100% scan efficiency to traditional 1D navigators, whereby the 3DSN scan was setup as a single shot scan and was called by the high resolution segmented multishot CMRA scan each cardiac cycle. We have shown in previous work that correcting all acquired data in foot-head (FH) and left-right direction (LR) can provide equivalent image quality to traditional gated scans (J Powell et al. 2013 Proc. ISMRM). Here we sought to investigate and compare the effect of correcting data in 3 dimensions (FH, LR and AP).

## Methods

**Figure 1:**
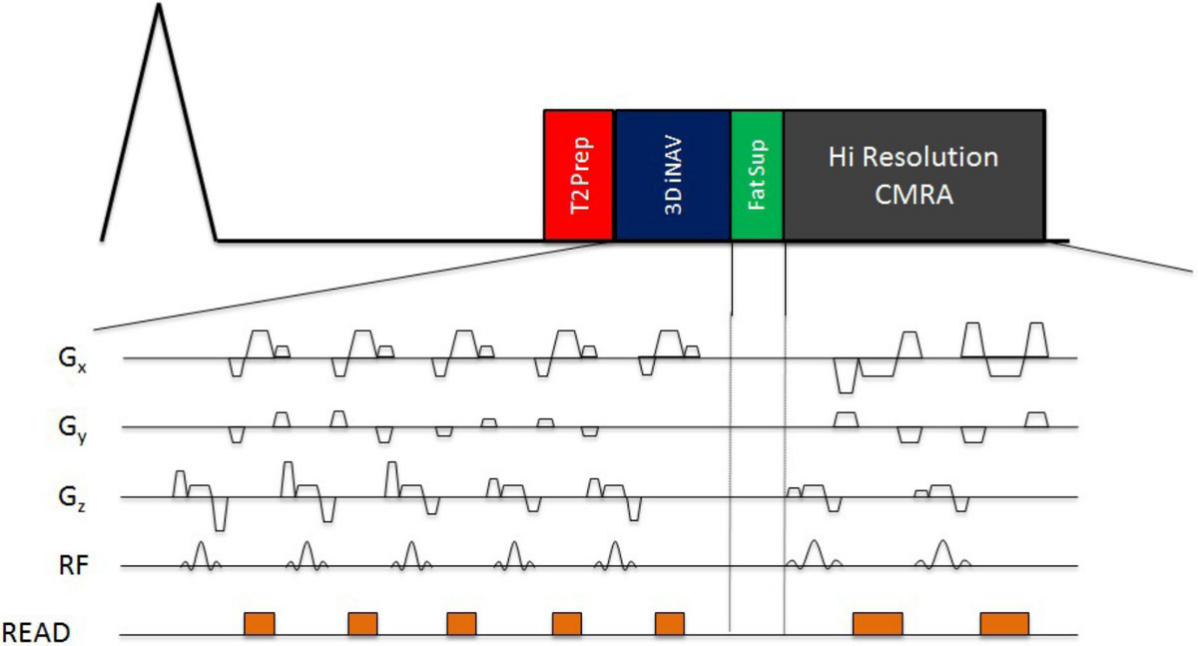
**In each R-R interval, we interleave two separately defined scans**. A single complete 3D iNav with it's own independent imaging parameters, and one set of segmented profiles of a high resolution CMRA scan.

The gradient echo 3DSN images with Cartesian k-space sampling were acquired in each R-R interval directly prior to the high resolution image acquisition by a simple function call that switched from the CMRA to the 3DSN scan (Figure 1). Each low resolution 3DSN volume is acquired at the same FOV as the high resolution gradient echo CMRA scan. The imaging parameters included a FOV of 300 × 300 mm, slab thickness of 80 mm, a spatial resolution of 1 × 1 × 2 mm, TR/TE = 5.3/2.6 ms and FA = 20°, sampling = Cartesian. 3DSN volumes were acquired in 81 ms using a total of 24 profiles, which was achieved with a spatial resolution of 5 × 10 × 10 mm, TR/TE = 3.4/1.7 ms and FA = 2°, and a SENSE factor of 3. For comparison, 1D navigator diaphragmatic gated whole heart datasets with identical parameters and a gating window of 8 mm was acquired. 4 healthy volunteers were scanned on a Philips 3T Achieva scanner (Philips Healthcare, Best, NL).

## Results

**Figure 2:**
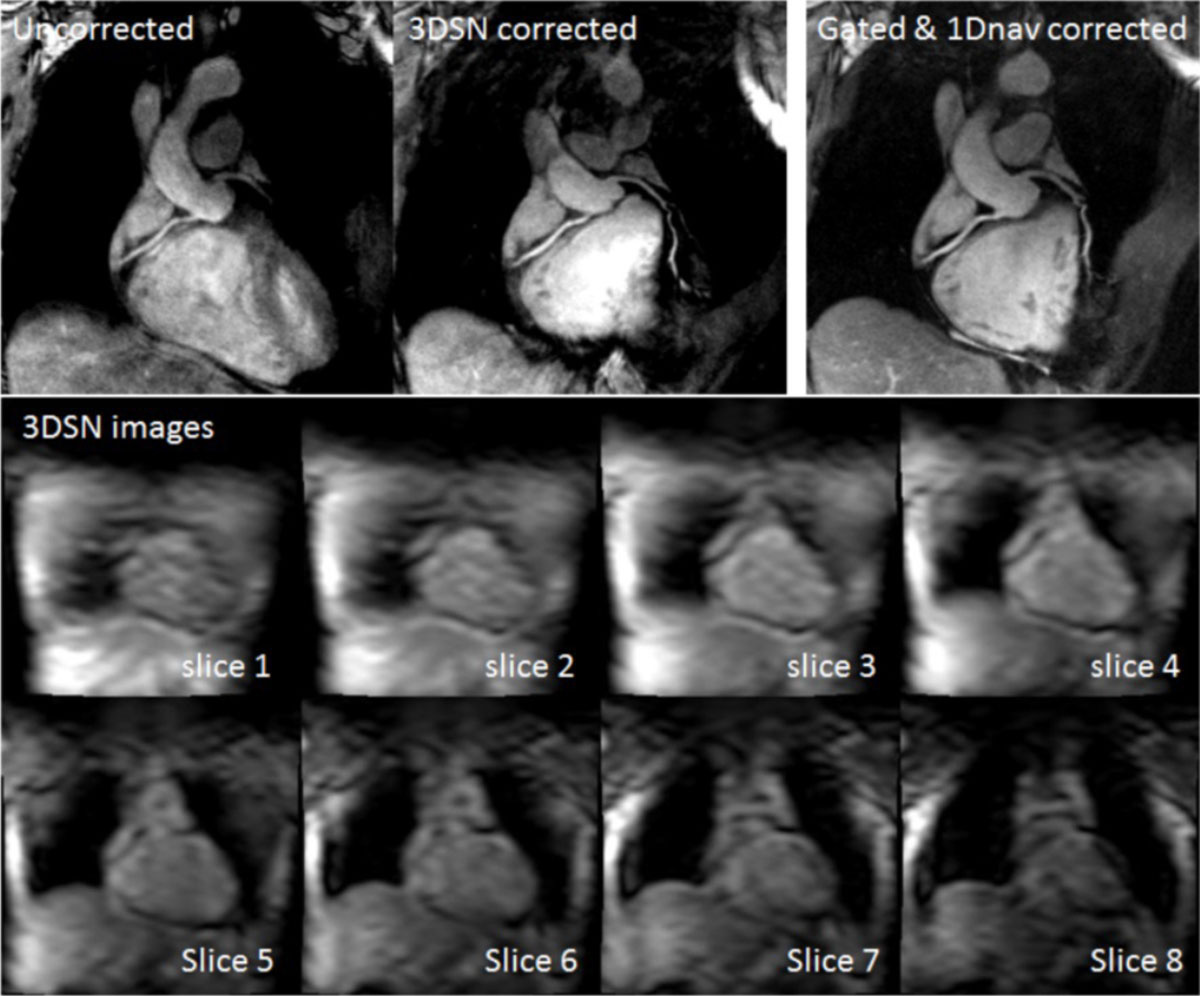
**In the uncorrected images the coronary arteries are blurred and indistinct**. After 3DSN correction there is a marked improvement in vessel sharpness, and much more of the vessel's length can be visualised. The 3DSN images show a complete 3D Volume acquired immediately prior to one set of high resolution CMRA profiles.

All scans were reconstructed successfully with 3DSN motion correction. Figure 2 shows an example set of reconstructed CMRA images and 3DSN images. It can be seen that the corrected images are much sharper and provide better vessel delineation than the uncorrected images, and are comparable to the reference images. The uncorrected images have a vessel sharpness of 59 +/- 0.07% that of the reference images, while the corrected images achieve vessel sharpness of 88 +/- 0.05% that of the reference images.

## Conclusions

3DSN corrected MRA images achieve comparable image quality compared to 1D navigated images. As data is not rejected outside of a gating window, the 3DSN scans are performed with an effective navigator efficiency of 100%, substantially reducing scan time. In subjects with significant cardiac motion in a non FH direction, 3DSN should lead to better results. Future work will focus on using 3DSN images to generate affine transformation data, and perform affine motion correction.

## Funding

This work was partially supported by a grant from Philips Healthcare, and a British Heart Foundation program grant (RG/12/1/29262).

